# Materials-oriented integrated design and construction of structures in civil engineering—A review

**DOI:** 10.1007/s11709-021-0794-9

**Published:** 2022-02-03

**Authors:** Xing Ming, John C. Huang, Zongjin Li

**Affiliations:** 1 Institute of Applied Physics and Materials Engineering, University of Macao, Macao SAR, 999078 China; 2 CHC Engineering, LLC, Fairfax, VA 20030 USA

**Keywords:** integrated design and construction, fiber-reinforced concrete, fiber-reinforced polymer, light-weight steel structures, digital fabrication, composites

## Abstract

Design is a goal-oriented planning activity for creating products, processes, and systems with desired functions through specifications. It is a decision-making exploration: the design outcome may vary greatly depending on the designer’s knowledge and philosophy. Integrated design is one type of design philosophy that takes an interdisciplinary and holistic approach. In civil engineering, structural design is such an activity for creating buildings and infrastructures. Recently, structural design in many countries has emphasized a performance-based philosophy that simultaneously considers a structure’s safety, durability, serviceability, and sustainability. Consequently, integrated design in civil engineering has become more popular, useful, and important. Material-oriented integrated design and construction of structures (MIDCS) combine materials engineering and structural engineering in the design stage: it fully utilizes the strengths of materials by selecting the most suitable structural forms and construction methodologies. This paper will explore real-world examples of MIDCS, including the realization of MIDCS in timber seismic-resistant structures, masonry arch structures, long-span steel bridges, prefabricated/on-site extruded light-weight steel structures, fiber-reinforced cementitious composites structures, and fiber-reinforced polymer bridge decks. Additionally, advanced material design methods such as bioinspired design and structure construction technology of additive manufacturing are briefly reviewed and discussed to demonstrate how MIDCS can combine materials and structures. A unified strength-durability design theory is also introduced, which is a human-centric, interdisciplinary, and holistic approach to the description and development of any civil infrastructure and includes all processes directly involved in the life cycle of the infrastructure. Finally, this paper lays out future research directions for further development in the field.

## 1 Introduction

Civil engineering has been an indispensable part of human lives since the beginning of civilization, creating the necessary infrastructure for human society [1]. It is the embodiment of human retrofitting and adaptation of the natural environment. Since the earliest practice at about 2000000 BC in Africa of stacking stones to hold branches in position [2,3], civil engineering has developed into a professional discipline that involves many sub-disciplines from different fields [4]. Thus, design and construction in civil engineering are complex processes, where material properties, structural forms, and even environmental effects should be holistically taken into consideration.

In civil engineering, materials and structures are two crucial components. As the base components for structures, materials are shaped into different elements such as beams, walls, columns, and frames. Materials and structures used in civil engineering have changed a lot since the earliest constructions. In the Stone Age, our ancestors used naturally existing caves and rock overhangs as shelters. Later, stone, clay, and wood were often used to build dwellings. But these dwellings were too simple to be regarded as formal constructions. Then,

*Article history: Received May 19, 2021; Accepted Jul 29, 2021*

natural building materials such as stone and wood were used to form walls, columns, and roofs in ancient Egypt and Greece. Constructions started to have standardized, symmetrical, and precise characteristics [5]. Buildings at that time were mainly used for living and protection purposes. The compatibility between materials and structures was far from being considered in construction processes. In Roman times, three different building materials were fully developed: 1) stone and masonry, 2) concrete, and 3) timber and metal [6], which expanded the variety of structural forms. At that time, structural forms followed material properties, i.e., optimal structural forms were selected that took full advantage of material properties. Thus, stone or masonry arch structures and various flexible timber structures and connections were extensively used. With the advent of the Industrial Revolution, building materials were able to be mass-produced [5]. Subsequent materials development has created man-made materials with better performance that have been integrated into civil engineering. On one hand, traditional structures and constructions can be made in new forms with these more performant materials, freeing structural forms from being highly dependent on the properties of materials used. On the other hand, new structures, such as steel structures, concrete structures, and reinforced concrete (RC), gradually replaced these traditional structures and constructions made with natural materials. New design methods and construction technologies were developed for these new structures, which facilitated a more scientific approach to design and construction than the traditional empirical method. Nowadays, composite materials, nanomaterials, and high-performance materials are flourishing with advanced theories and manufacturing technologies [7–12]. Structures can now be made in various forms according to demand, allowing for ultra-high buildings, long-span bridges, undersea tunnels, and other special-purpose structures [13–17]. We can even customize the properties of various materials used in a structure as needed. Although more materials and structural forms are available in civil engineering, it is still a challenge for young engineers to work out how to integrate them.

In this paper, we review how to implement Material-oriented integrated design and construction of structures (MIDCS) in a civil engineering context, drawing upon some relevant real-world scenarios, to give some inspiration and guidance to readers interested in implementing MIDCS themselves. We first outline the definition and principles of MIDCS, and then present an in-depth discussion and review of MIDCS based on some typical practices in existing structures, from past to present. We subsequently discuss advanced methods and technologies useful for effectively implementing MIDCS in civil engineering, summarize our findings, and recommend directions for further research.

## 2 Definition and principles of material-oriented integrated design and construction of structures

MIDCS is a long-standing and classic approach intentionally or unintentionally used in many engineering aspects. Briefly, MIDCS in civil engineering refers to designing and constructing a building or infrastructure while considering material properties and structure functions in an integrated fashion. The intertwined relationship between materials and structures is illustrated in Fig. 1. The base of the bottom pyramid represents materials, with four corners each representing synthesis, microstructures/composition, properties, and performance. The top of the inverted pyramid represents four structural engineering aspects: safety, durability, serviceability, and sustainability. Material and structural engineering both have to meet the end-use needs and constraints of the final structure. As shown in Figs. 1 and 2, MIDCS includes two approaches. 1) From bottom to top: this is a bottom-up design approach. In this approach, the properties of existing materials determine the possible structural forms. This approach is the traditional method employed by builders and engineers in the centuries past. 2) From top to bottom: this is a top-down approach. In this approach, structural engineers need to translate targeted structural performance to desired materials properties that may include mechanical properties, microstructure, or macrostructure; material engineers need to invent new composite materials or customize the existing materials to meet the desired material properties [18–20]. In other words, the desired performance of structures guides the material's design and development. This top-down approach is a relatively new engineering paradigm in civil engineering, which is made possible by advancing material engineering and construction

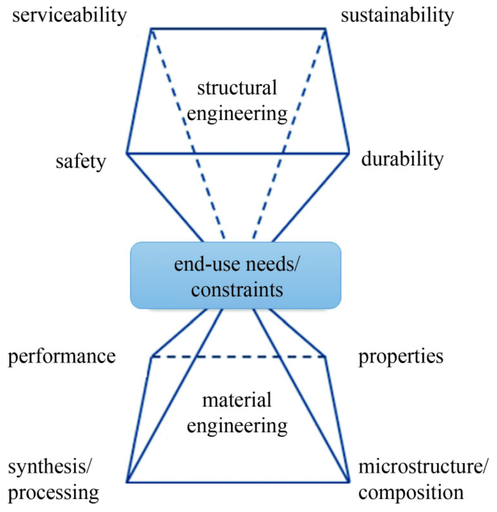

**Fig. 1** The intertwined relationship between materials and structures [18].

technologies in recent decades. Advanced design methods and construction technologies can bridge the "top to bottom" or "bottom to top" paths between materials and structures shown in Fig. 2. In the past, only the first approach of MIDCS was usually considered when constructing civil infrastructures, which caused suboptimal materials and structures to be used and decreased their service lives. Nowadays, both approaches of MIDCS should be considered to develop high-performance structures.

## 3 Practical examples of material-oriented integrated design and construction of structures

### 3.1 Timber-based seismic-resistant structures

Timber (wood) is a kind of traditional building material. It is widely used in various civil engineering structures for different functions because of its availability, relatively low cost, ease-of-use, eco-efficiency, attractive aesthetics, good compatibility with other materials, and decent durability. However, there are many substitute building materials for timber, such as concrete, steel, and composite materials [1,25]. Throughout timber's long history as a building material, applying integrated design to timber has been key to preserving ancient timber structures and achieving a long service life. In the following sections, we first examine the microstructures and properties of timber. Then we focus on ancient timber structures, showcasing the ingenious designs and construction processes used with timber to produce seismic-resistant structures.

#### 3.1.1 Properties of timber

Timber is a natural, renewable, and sustainable product mainly from the trunks of trees [1]. As shown in Fig. 3 [26], moving from outside to inside, it consists of bark, cambium, xylem, and pith. It is chemically composed of cellulose, lignin, hemicellulose, extractives, and ash-producing minerals [1]. The compositions, characteristics, and functions of the different timber parts are listed in Table 1 [1,27]. Timber is a type of natural organic polymer composite material. Its highly ordered microstructure demonstrates intrinsic anisotropic physical and mechanical properties. Typically, the three important directions related to the anisotropic properties of timber are the tangential, radial, and longitudinal axes. The properties of timber along different directions are described in these valuable references [1,27,28]. The compressive and tensile strengths are higher along the longitudinal direction than their counterparts along the radial direction. This is because of cellulose structural elements' greater bearing capacity along the grain direction (longitudinal direction). Therefore, when timber is used to resist tension or compression, external loads should be applied along the longitudinal direction to take full advantage of its high bearing capacity. Similarly, when timber is used to resist shear forces, loads should be applied along the radial direction rather than the

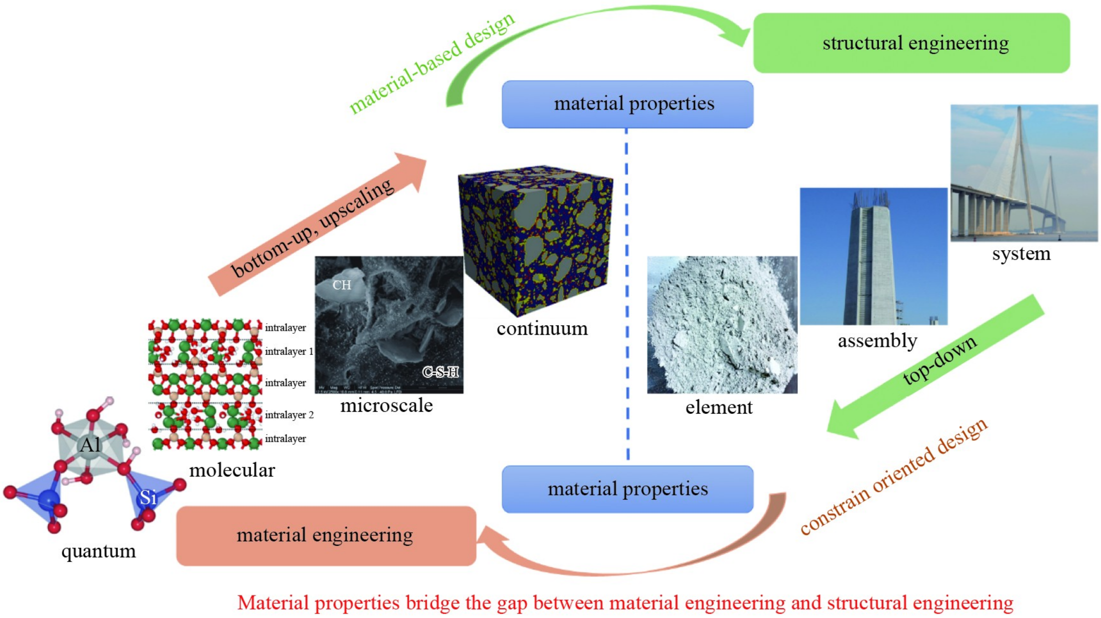

**Fig. 2** Schematic illustration of the concepts of MIDCS in civil engineering (Reprinted from Cement and Concrete Research, 141, Zuo Y, Ye G, GeoMicro3D: A novel numerical model for simulating the reaction process and microstructure formation of alkali-activated slag, 106328, Copyright 2021, with permission from Elsevier). Notes: the images from quantum to continuum are from Refs. [21–24]; the images for structural engineering are from Wikipedia. All these images are reproduced by the authors.

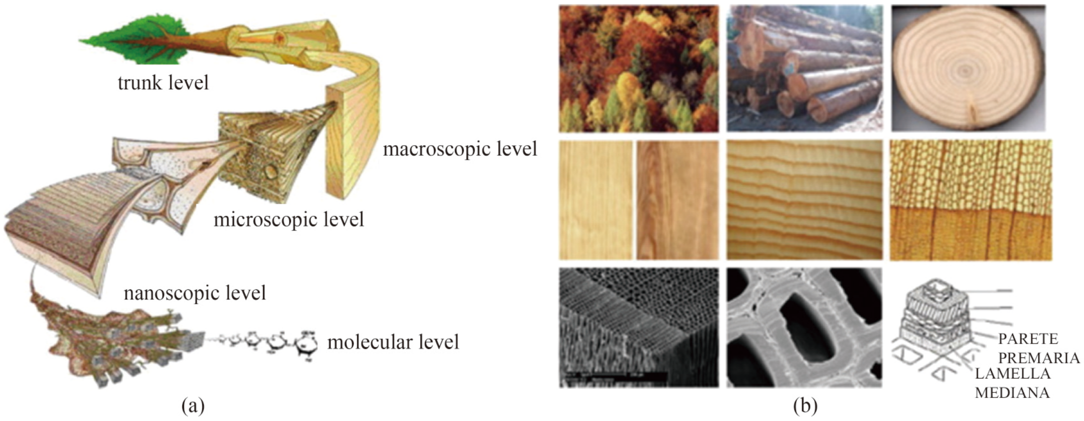

**Fig. 3** (a) Levels present in timber; (b) variability of timber at different scales [26] (Reprinted from Construction and Building Materials, 97, Marzi T, Nanostructured materials for protection and reinforcement of timber structures: A review and future challenges, 119–130, Copyright 2015, with permission from Elsevier).

**Table 1** Compositions, characteristics, and functions of different parts of timber [1,27]

| series | part | presence (%) | characteristics | functions |
|---|---|---|---|---|
| physical composition | bark | 7–20 | A dead and corky outer layer with variable thickness and a growth inner layer | √ Label of tree species √ Medicinal material √ Origin of fibers and thermal insulation material |
| | cambium | N/A | A thin layer of cells with meristematic ability between bark and xylem | √ Origin of bark and xylem |
| | xylem | N/A | Low moisture, dark, hard, dead, and high-density for heartwood; opposite characteristics for sapwood | √ Heartwood provides structural strength and is resistant to decay; the origin of timber √ Sapwood is the storehouse for starches and a pipeline for sap, and it is not durable |
| | pith | N/A | The central core of the tree with variable structure, size, and color | √ No engineering applications |
| chemical composition | cellulose | N/A | A linear polymer with highly ordered strands (also called fibrils) | √ Source of large structural elements and cell walls of wood fibers |
| | lignin | 23–33 in softwood, and 16–25 in hardwood by weight | An intercellular material that glues tubular cells together | √ Source of longitudinal shear strength |
| | hemicellulose | 15–20 in softwood and 20–30 in hardwood | A sugar-based material with polymeric units | √ Cross-links cellulose fibrils, strengthening cell walls |
| | extractives | 5–30 | Nonstructural elements containing coloring, essential oils, etc. | N/A |
| | minerals | 0.1–3 | Calcium, potassium, etc. | N/A |

Note: N/A means the data is not applicable for this item.

longitudinal direction because of the poor bonds between tubular cells. However, the tubular cells are good at dissipating energy, leading to good damping and isolation effects, which will be discussed in the next section.

### 3.1.2 Towards seismic-resistant structures

As discussed above, timber structures can survive moderate earthquakes because of their intrinsic damping and isolation abilities. However, these abilities are insufficient to dissipate sufficient energy during strong earthquakes and avoid sudden collapse induced by earthquakes because timber is still brittle in tension, bending, and shear parallel to the grain (in the longitudinal direction) [29]. Therefore, special timber connections are used to achieve the high flexibility and ductility needed to dissipate the energy of moderate earthquakes and prevent the timber structure from being damaged under seismic forces [30–34]. Figure 4 shows some typical ancient timber structures [35–37], and Fig. 5 shows the components of an ancient timber hall-style structure [38]. Ancient Chinese and Japanese timber structures mainly consist of four parts from bottom to top, i.e., stylobate (plinth), column frame, bracket set, and

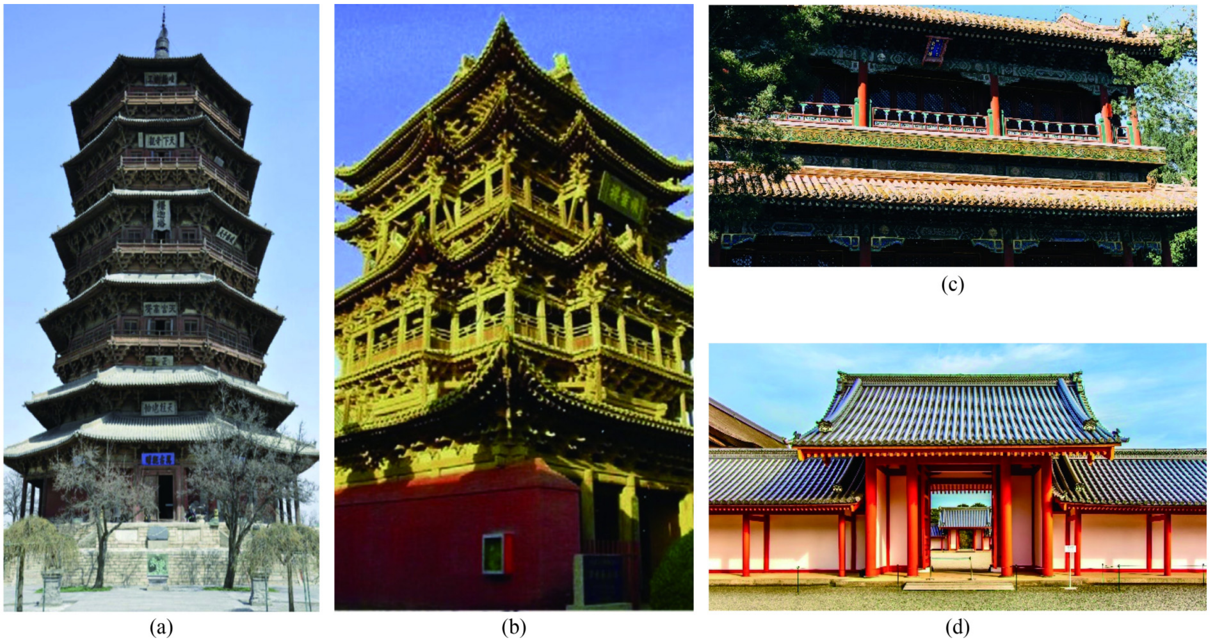

**Fig. 4** Typical ancient timber structures: (a) Yingxian Wood Pagoda [35]; (b) Feiyun Pavilion [35]; (c) the Imperial Palace in China; (d) the Imperial Palace in Japan (Reprinted from Construction and Building Materials, 105, Qiao G F, Li T Y, Chen Y F, Assessment and retrofitting solutions for an historical wooden pavilion in China, 435–447, Copyright 2016, with permission from Elsevier).

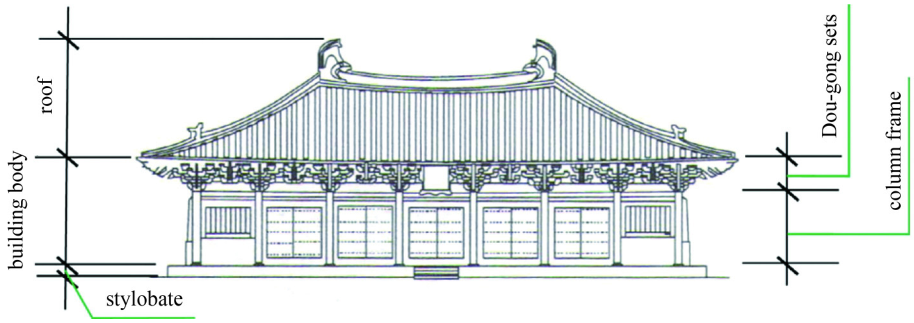

**Fig. 5** Components of an ancient timber hall-style structure [38] (Reprinted from Engineering Structures, 156, Chen J Y, Li T Y, Yang Q S, Shi X W, Zhao Y X, Degradation laws of hysteretic behaviour for historical timber buildings based on pseudo-static tests, 480–489, Copyright 2018, with permission from Elsevier).

**Table 2** Timber connections and related functions for seismic resistance [28,30,36,39,40]

| connection | characteristic | function |
|---|---|---|
| dougong | elastic and self-locking when loaded | damping effect |
| mortise and tenon | semi-rigid | damping effect |
| column base | flexible | damping and isolation effects |

roof, although they may come in different styles for different functions [31]. Table 2 summarizes the connections between the various parts and their functions for seismic resistance [28,30,36,39,40]. In general, the seismic resistance of an ancient timber structure is realized by the damping and isolation effects of the timber connections between different parts.

Typically, while the stylobate is anchored in the foundation, the column is simply laid on top of the stylobate [39]. This connection between stylobate and column produces both isolation and damping effects that reduce the damage of earthquakes. The isolation effects stem from the slidable interface between stylobate and column, which reduces the effect of an earthquake on superstructures. The maximum force experienced by the superstructures will not be higher than sliding friction between stylobate and column, producing the isolation effect of this special timber connection. The flexibility of the column above the connection reduces the effective acceleration, relative velocity, and maximum relative displacement, producing the damping effect of this connection [28,31,41].

The upper part of the column and beam are typically connected with mortise and tenon. The slip and rotation between the mortise and tenon under lateral loads help dissipate the energy of an earthquake [36,42–45]. Moreover, the column can be regarded as a self-centering structure under these special connections, thus further producing a damping effect [31,46]. Dougong is the main element of the bracket set and connects the column and beam/roof. It has many layers, and the friction between layers can also dissipate the energy of an earthquake to produce a damping effect [31,40].

In summary, ancient timber structures in China and Japan have moderate seismic resistance via various damping and isolation effects. It should be noted that the special forms for seismic resistance of timber structures are related to the special properties of timber, such as its tubular cell structure, ease of processing, low modulus, and anisotropic behavior. Thus, ancient timber-based seismic-resistant structures are examples of building construction driven by material properties, which is one of the important aspects of MIDCS. Additionally, designs driven by end-use demands of structures can be seen in some modern timber structures that are made using advanced processing methods and technologies (see Fig. 6) [26]. In these examples, the engineered timbers act as the main tension or compression elements and provide decorative effects.

### 3.2 Masonry arch structures

Masonry is also an old building material and is widely used in various structures worldwide [1,47,48]. Although it has been transformed from a traditional load-bearing form into a non-load-bearing role with multiple functions (such as fire protection, thermal and sound insulation, weather protection, and sub-division of space) in the past several decades [49–52], it still plays an important role in building constructions. It cannot be completely replaced by concrete, steel, or even composite materials [51]. Here, we mainly focus on masonry arch structural forms to present the concept of MIDCS in the practice of masonry construction.

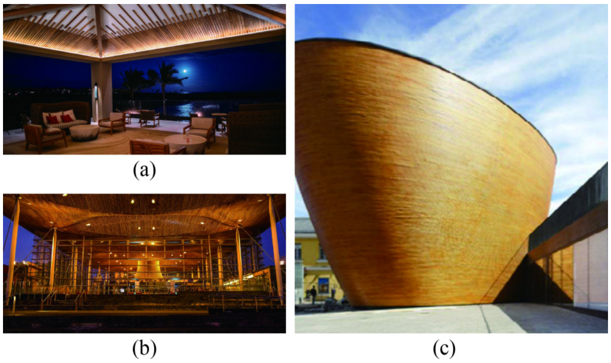

**Fig. 6** Modern timber structures: (a) timber roof; (b) National Welsh Assembly; (c) Kamppi Chapel of Silence in Helsinki [26] (Reprinted from Construction and Building Materials, 97, Marzi T, Nanostructured materials for protection and reinforcement of timber structures: A review and future challenges, 119–130, Copyright 2015, with permission from Elsevier).

#### 3.2.1 Properties of masonry

A masonry structure is usually formed by masonry units and mortar [1,51]. Masonry units make up the main masonry structure, accounting for more than 93% of its volume. A unit can be either a “brick” or a “block” depending on its size, and is made up of concrete, clay, calcium silicate, natural stone, etc. Masonry units come in the forms of solid, perforated, or hollow units [1,51]. A masonry structure can have many functions given the appropriate raw materials and forms. For example, the compressive load-bearing function of a masonry structure can be realized by using solid concrete blocks or clay bricks [53–55]. The thermal and sound insulation functions can be achieved with aerated concrete blocks [56]. Aesthetic effects can be attained by controlling the color of clay bricks used [51]. Another important part of masonry is mortar, although it only accounts for 7% of the total volume of a masonry structure. The mortar is usually a mixture of cement, sand, lime, and water [1,51]. Also, chemical admixtures may be used to control the workability of the mortar. Mortar should be carefully produced to satisfy the functional demands of a masonry structure.

Just like timber, masonry is also an anisotropic material [47,57,58]. It can better bear external loads perpendicular to the direction of the mortar joint. The compressive strength of a masonry unit is much higher than its tensile or flexural strength. Hence, compressive strength should be the predominant factor in designing a load-bearing masonry structure. However, horizontal shear forces or lateral pressure from wind and earthquakes should also be considered wherever masonry is used [50]. Thus, the design of masonry structures needs to deal with the incompatibility between end-use demands of the actual structures and intrinsic mechanical properties of masonry. The examples described below perfectly solve this incompatibility and demonstrate the power of MIDCS when applied to masonry and its related structures.

#### 3.2.2 Towards arch structures

The arch structure is the most effective form to avoid generating tensile, and shear stresses inside of a structure. It is a typical example of integrated design: it uses a structural form that takes full advantage of the high compressive strength of masonry. The first masonry structure with arches may date back to 4000 BC when natural stone or clay bricks were used to build corbel arches for underground structures [48]. Later, arch technology was rapidly developed by the Roman Empire. The arch forms can be found in various kinds of masonry structures such as bridges, roofs, and sewers. The Romans also produced new forms by crossing arches together, creating cross vaults [59]. Typical masonry arch structures can be seen in Fig. 7 [59].

Take the Zhaozhou Bridge (also called Anji Bridge) as an example (Fig. 8). The Zhaozhou Bridge is a stone arch bridge about 50 m long with a central span of 37.37 m. The arch covers a circular segment less than half of a semicircle (84°) and with a radius of 27.27 m. The central arch is made of 28 thin, curved limestone slabs joined with iron dovetails. This allows the arch to adjust to shifts in its supports and prevents the Bridge from collapsing even when a segment of the arch breaks. The Bridge has two small side arches on either side of the main arch. These side arches serve two important functions. First, they reduce the total weight of the Bridge by about 15.3%. Second, when the Bridge is submerged during a flood, they allow water to pass through, reducing the Bridge structure's lateral forces. In this arch bridge, the vertical loads on the bridge are converted into internal compressive stress along the axis of the side arches and then the main arch, fully utilizing the excellent compressive strength of layered stone rather than its poor tensile force carrying ability. With its stone arch structures, the Zhaozhou Bridge perfectly reflects the bottom-up approach of MIDCS.

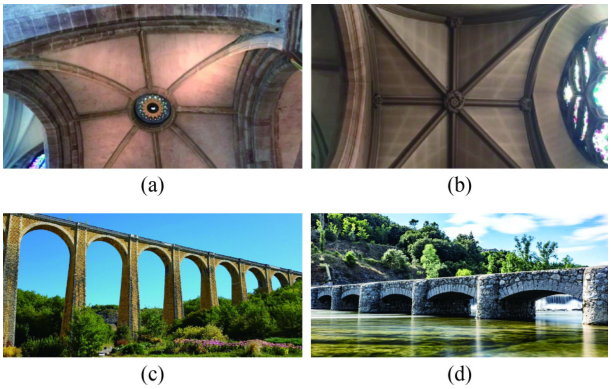

**Fig. 7** Typical masonry arch structures: (a) and (b) are cross vaults in Scotland [59] (Reprinted from Engineering Structures, 180, Bertolesi E, Adam J M, Rinaudo P, Calderón P A, Research and practice on masonry cross vaults—A review, 67–88, Copyright 2019, with permission from Elsevier); (c) masonry arch bridge in France; (d) stone arch bridge in France.

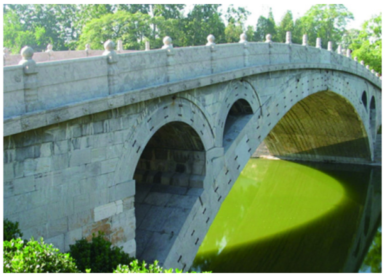

**Fig. 8** The Zhaozhou Bridge (image from Wikipedia).

### 3.3 Steel-based long-span bridges

The steel used in civil engineering greatly advanced the structural forms of modern constructions and fostered the development and construction of high-rise buildings and large-span bridges [15,16,60,61]. Additionally, these modern steel structures demanded better material properties, which accelerated the production of high-performance steel materials [5,15]. Currently, steel and its alloys are widely used in civil engineering in the forms of structural elements, fastenings, and decorative materials because of their high tensile strengths, favorable physical properties, and good casting abilities [1,62].

#### 3.3.1 Properties of steel

The mechanical properties of steel are of great importance for its application in bridge engineering. The steel components used in long-span bridges mainly carry tension and ensure a ductile failure mode because of their high tensile strength and good ductility. These mechanical properties can be improved further by incorporating alloying agents. For example, incorporating manganese will increase the ductility, toughness, and abrasive resistance of steel. Chromium helps improve the corrosion resistance of steel [1,63–65]. There are two other methods of modifying the mechanical properties of steel: 1) cold working accompanied with aging treatment, and 2) heat treatment [1,66]. The first method usually refers to cold drawing and cold rolling of steel at an ambient temperature, then letting processed steel products stand for several days or several hours at 100–200 °C. This improves the tensile strength but sacrifices plasticity and toughness to some extent. The second method is heating the steel in a special regime to change its hardness, ductility, plasticity, toughness, and strength. It usually includes a series of methods such as annealing, normalizing, hardening, and tempering [1].

In general, steel has the following advantages for its use in civil engineering structures: 1) homogeneous properties; 2) excellent physical-mechanical properties; 3) good machinability; 4) easy connection with other elements by welding or fastening; 5) customizable mechanical properties via alloying, cold working, or heat treatment. Due to these advantages, steel is widely used in long-span structures such as cable-stayed bridges and suspension bridges [15,67].

#### 3.3.2 Towards cable-stayed and suspension bridges

Cable-stayed bridges and suspension bridges are the two main forms of long-span bridges around the world [16]. Tables 3 and 4 present an overview of the world's top five cable-stayed bridges and suspension bridges.

**Table 3** Top five cable-stayed bridges in the world

| rank | name | main span (m) | year opened | country | main materials | image |
|---|---|---|---|---|---|---|
| 1 | Russky Bridge | 1104 | 2012 | Russia | RC bridge tower and galvanized PSS cables |  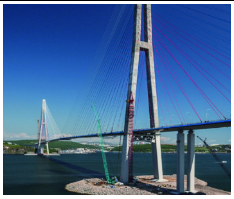 |
| 2 | Hutong Yangtze River Bridge | 1092 | 2020 | China | RC bridge tower |  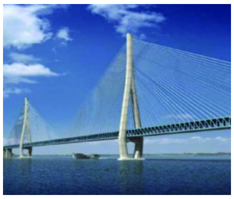 |
| 3 | Sutong Yangtze River Bridge | 1088 | 2008 | China | RC bridge tower and steel box girder |  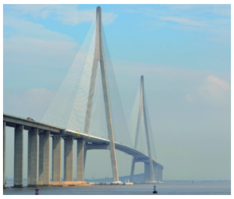 |
| 4 | Stonecutters Bridge | 1018 | 2009 | China | Concrete and stainless-steel skin for the tower, steel for the main span, concrete for side spans |  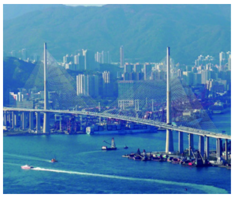 |
| 5 | Edong Yangtze River Bridge | 926 | 2010 | China | RC bridge tower, steel box girder, and galvanized PSS cables |  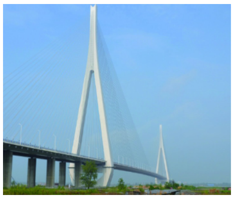 |

Note: PSS means parallel strand stay. Bridge data is from Wikipedia and the image of Hutong Yangtze River Bridge is from Ref. [16] (Reprinted by permission from Springer Nature Customer Service Centre GmbH: Springer Nature, Frontiers of Structural and Civil Engineering, Design and construction of super-long span bridges in China: Review and future perspectives, Huang W, 2020).

Compared with traditional masonry bridges, these modern bridges usually exhibit much longer spans, which are necessary to cross wide rivers and allow large ships to pass under the bridge. These modern bridges are also important symbols of a country's scientific and technological prowess [16]. Cable-stayed bridges and suspension bridges typically consist of cable towers, main girders, and stayed cables (for cable-stayed bridges) or suspension cables (for suspension bridges). The cable towers can be made of either (prestressed) RC or steel, and the cables are high-strength steel wires [16,68]. Steel is used in tension predominant structural elements of both cable-styled bridges and suspension bridges because of the excellent mechanical properties of steel discussed above.

The design and construction of long-span bridges also promote the development of high-performance steel. For example, to meet the needs of high strength, structural stiffness, and flexible geometries induced by the increase of bridge spans and the development of multi-function combined bridges, high strength bridge steel such as Q420q and Q500q have been successfully developed and used in the Dashengguan Yangtze River Bridge and the Hutong Yangtze River Bridge, respectively. Functional coatings, high-strength steel wires, and other advanced technologies are commonly used in steel cables to improve their load-bearing capacity as well as their corrosion resistance [15].

In summary, steel is widely used in cable-stayed bridges and suspension bridges due to its excellent tensile properties. Also, the development and construction of these high-performance structures further facilitate the production and utilization of high-performance steel, such as high-strength steel and anti-corrosion steel, in civil engineering structures. This, in turn, allows for more development of high-performance steel structures. This

**Table 4** Top five suspension bridges in the world.

| rank | name | main span (m) | year opened | country | main materials | image |
|---|---|---|---|---|---|---|
| 1 | Akashi Kaikyō Bridge | 1991 | 1998 | Japan | Steel bridge tower, steel truss, and high strength galvanized cables |  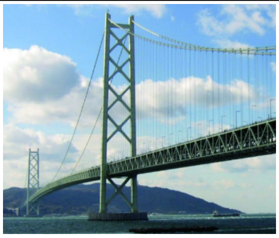 |
| 2 | Yangsigang Yangtze River Bridge | 1700 | 2019 | China | RC bridge tower, steel truss, and high strength galvanized PPWS cables |  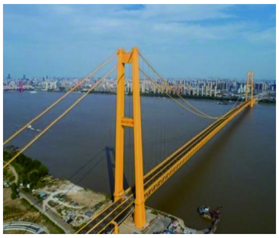 |
| 3 | Nansha Bridge | 1688 | 2019 | China | Steel box girder and high strength galvanized cables |  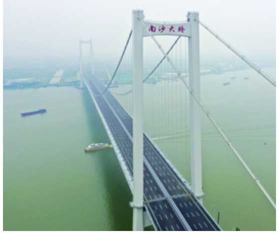 |
| 4 | Xihoumen Bridge | 1650 | 2009 | China | RC bridge tower, steel box girder, and high strength galvanized PPWS cables |  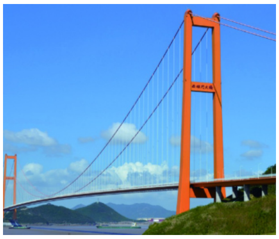 |
| 5 | Great Belt Bridge | 1624 | 1998 | Denmark | RC bridge tower, steel box girder, and steel cables |  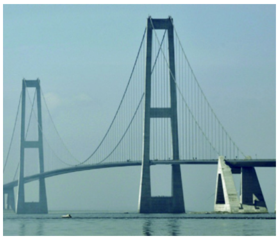 |

Note: PPWS means prefabricated parallel wire strands. Bridge data is from Wikipedia and Ref. [69]. The images of Yangsigang Yangtze River Bridge and Nansha Bridge are from Wikipedia.

mutually promoting relationship illustrates the concepts of integrated design and construction for steel.

### 3.4 Lightweight steel structures in the computational age

Lightweight steel structures are usually made from cold-formed steel (CFS) products such as wall studs and floor/roof decks. CFS products are shaped at ambient temperature from steel sheets, strip plates, or flat bars by roll forming machines, press brakes, or bending brake operations. They can be mass-produced in large quantities and at high speeds with consistent quality. A typical automated rolling machine can run at a speed range of 23–46 m/min, and the size of products can be as small as a 20 mm wide cold-rolled channel section to as big as a 915 mm wide roof deck section. In addition, CFS products possess several advantages over other construction materials: lightweight, high strength, high rigidity, dimensional stability, durability, non-combustibility, and recyclability [70].

#### 3.4.1 Properties of cold-formed steel

The process of cold-forming changes the mechanical properties of steel because certain parts of the material undergo plastic deformation. As a result, the average yield strength of steel over the entire cross-section increases by approximately 15% to 20%, depending on the amount of deformation introduced. The cold-forming process increases the material strength and, more importantly, the final product's strength and rigidity (stiffness). As shown in Fig. 9, a thin, flat sheet of steel cannot support much weight, but if it is roll-formed into a corrugated sheet, the folds act as stiffeners and can increase the strength and rigidity of the same sheet many times over. For example, when a 1.0 mm thick flat sheet of steel is folded into a 38 mm deep corrugated roof deck, the finished product's bending strength and stiffness are increased to roughly 100 times and 4600 times that of the original flat sheet of steel, respectively. Due in part to this unique characteristic, about 40% of the steel used in construction in the United States is CFS, ranging from roof/floor decks to wall studs/panels to window/door frames.

### 3.4.2 Towards digital design, fabrication, and construction

Lightweight steel structures are usually manufactured at a factory and assembled on-site. A combination of hot-rolled and cold-formed steel framing components, metal roofing systems and wall panels of varying materials are used to provide a complete building envelope system that is air-tight, energy-efficient, economical, and above all, designed to suit particular user specifications. Computer-aided analysis, design, and detailing of lightweight steel structures provide a complete and accurate bill of materials, down to the exact number of bolts and screws needed to complete the project, thereby eliminating or minimizing construction waste. Due to their low cost and fast construction schedule, lightweight steel structures are widely used for small industrial and manufacturing facilities, retail stores, warehouses, storage units, job site trailers, and shipping containers. A recent example of a lightweight steel structure application is an instant hospital constructed in 10 d in Wuhan, China, during the Covid-19 pandemic. With most of the building components already prefabricated, the field assembly of the two-story, 1000-bed Huoshenshan Hospital took only 4 d after infrastructure and concrete floors were in place. The entire hospital was composed of hundreds of container-house-style modular units with easy-to-grab lifting holes, a slightly elevated floor to keep rainwater out, and insulated wall sandwich panels. This is an excellent example of integrated design and construction in a "top to bottom" approach, with time being the most critical constraint. The design selection of a prefabricated lightweight steel structure aided the fabrication process, expedited field installation, and met the stringent end-use requirements.

Besides its conventional uses, CFS has many unique applications, including the on-site extrusion of building

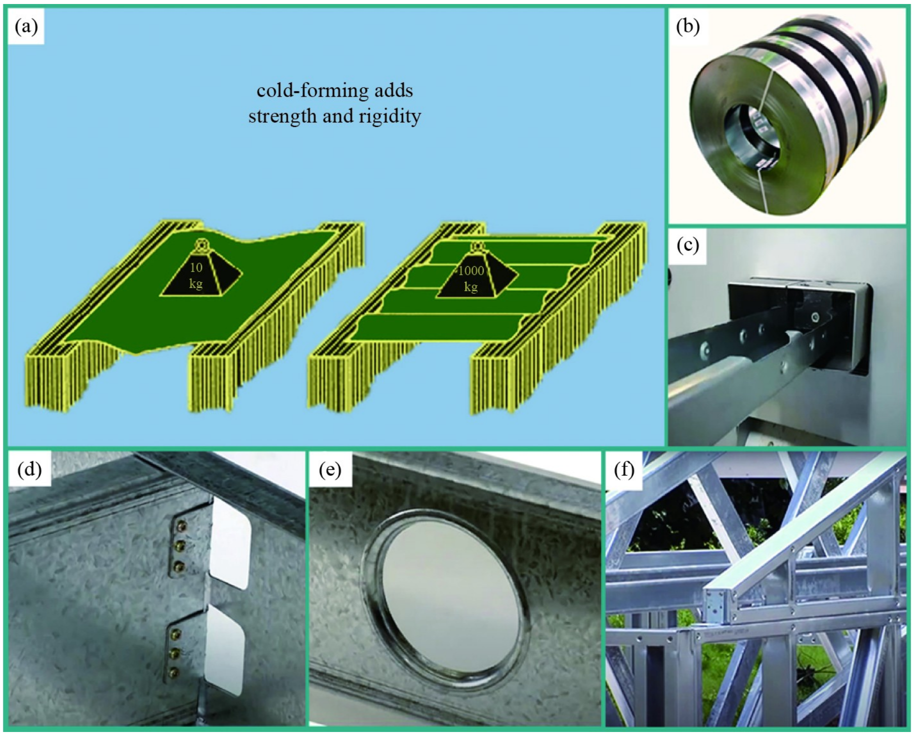

**Fig. 9** Cold-formed steel: (a) effects of cold-forming; (b) steel coils before cold-forming; (c) steel stud coming out of a roll-forming machine; (d) steel floor joist end connection; (e) pre-punched and stiffened hole in steel joist; (f) steel stud wall and roof framing. Notes: Components shown on (c), (d), (e), and (f) are all produced in CFS mobile factories. See Fig. 10 for the schematic diagram of a CFS mobile factory.

components on remote sites or faraway islands. Since CFS originally comes in a coil with a diameter of about 1.5 m and a width of about 0.15 to 0.5 m, it is very compact and can be easily transported without taking up a lot of cargo space. Steel coils can be fed into a roll-forming machine housed in a modified standard shipping container (a mobile factory), which means it can be shipped, trucked, or airlifted into any location and made operational within 24 h after delivery. The production speed of structural components using a CFS mobile factory can reach up to 700 m per hour. In addition, the mobile factory is fully computer-controlled, so every piece of framing produced is numbered and coded, making the framing easily assembled on-site by local workers (see Figs. 9(c) and 9(f)). A single steel coil can provide enough material to build a single-story house of approximately 5 m × 10 m in size, including roof trusses. This approach can speed up the construction process and make it possible to build structures with desired materials that are not cost-effective to transport due to long shipping distances. Figure 10 shows a schematic diagram of the digital design, fabrication, and construction of a cold-formed steel building using a roll-forming machine in a CFS mobile factory.

Compared with the mass production of CFS components in a factory setting, the customized digital on-site fabrication process makes it easy for contractors to practice lean construction, a concept similar to lean manufacturing. Lean construction aims to improve construction performance by eliminating waste and saving cost for customers, contributing to the built environment's sustainable development. Figure 9(d) shows a lean connection to punched shear tabs at the end of the steel floor joist. The pre-punched hole for ductwork has a stiffened edge as shown in Fig. 9(e), which is very hard to achieve for a field cut hole. These component fabrication details are examples of altering material's mechanical properties at the macrostructural level in a

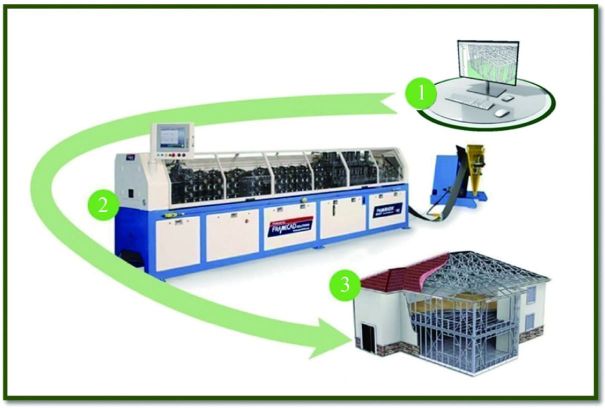

**Fig. 10** Schematic diagram of digital design, fabrication, and construction of a cold-formed steel building through a CFS mobile factory.

"top to bottom" approach. Because every piece of framing component is numbered and coded in this customized digital fabrication, this "top to bottom" integrated design and construction approach also paves the way for future automated building construction by robots, similar to the current practice of using manufacturing robots in the automotive industry.

### 3.5 Fiber-reinforced cementitious composite structures

Composite materials are man-made products engineered to have various desirable material properties. A composite material usually consists of two or more constituents with different physicochemical properties. The combined product, a composite material, is expected to perform better than the individual constituent materials [1]. Since the earliest use of straw- and mud-based composites in building shelters in Egypt [1], composite materials have come a long way in civil engineering. Nowadays, engineered wood and RC are typical composites used in civil engineering. Here, we focus on another composite material developed recently, fiber-reinforced cementitious composite (FRCC). It is a mixture of discontinuous fibers and cementitious composite [5]. Although it has similar properties and applications with traditional concrete, it does have some differences from RC. Recently, FRCC research has developed into new branches such as engineered cementitious composites (ECC) and their functionalized products (self-healing ECC, self-cleaning ECC, etc.) [10], ultra-high-performance fiber-reinforced cementitious composites (UHPFRCC) [71–73], and hybrid fiber-reinforced cementitious composites (HFRCC) [74–76] based on the Performance-Driven Design Approach.

#### 3.5.1 Properties of fiber-reinforced cementitious composites

FRCC is a typical composite material driven by structural performance. Superplasticizer and other mineral admixtures are used to improve the matrix's molding performance and rheological properties, allowing the fibers to be incorporated and homogeneously dispersed in the matrix. FRCC can demonstrate strain-hardening responses under tensile stress when the properties of the fibers, matrix, and the interfaces between them are adequately designed [5]. The fibers embedded in the matrix are the primary tensile stress-bearing elements after the material cracks. Recently, it has been found that aligning the embedded fibers along the tensile stress-dominated direction can improve performance even under high temperatures [77]. Based on the tensile responses of FRCC, it can be roughly divided into ordinary FRCC and high-performance FRCC (such as ECC and their functionalized products) [5,10]. Although both classes of FRCC can improve the toughness, peak strain, etc., of cementitious composites under compression [10], they exhibit different responses to tensile stress. In the past, maximizing compressive strength was the main reason for integrating structure construction with material design, so typical FRCC with similar compressive properties as high-performance FRCC, but less expensive, was more widely used. However, with the increasing demands on high-performance structures nowadays, the tensile properties, ductility, and toughness of materials are also being considered in MIDCS. High-performance FRCC exhibits desirable toughness and strength with low fiber content (e.g., ECC) and is now being used in modern high-performance structures [10]. Besides, these high-performance FRCC can be further engineered to have a high compressive strength higher than 150 MPa (UHPFRCC) [78] to meet the demands of designing ultra-high buildings. Real applications of ECC and UHPFRCC in structural elements and being repairing materials for strengthening damaged structural elements show that they possess favorable properties of both cementitious composites and fibers. On one hand, they can be sprayed or extruded, making them easy to apply [79,80]. On the other hand, they can bear both compressive and tensile stress as well as environmental load (good durability) when reasonably designed [10,81,82] and even handle dynamic loads [10,83,84]. Thus, they have a wide application in civil engineering.

#### 3.5.2 Towards high-performance structures

In civil infrastructures, successful real applications of ECC include composite bridge decks when reinforced by steel rebars, tunnel linings, link-slabs for bridge decks and pavement, coupling beams for high-rise buildings, and even dampers for seismic-resistant structures [10]. Here we mainly focus on the last three items to show why ECC can be used in these areas.

A bridge deck link-slab is a typical example of using ECC to fulfill structural demands. A typical link-slab for bridge decks usually possesses adequate moment capacity, good cracking resistance, and excellent hinge action to follow the original bridge span design. These end-demands of link-slab require that materials used for the link-slab have excellent toughness, ductility, and cracking-resistance and have a relatively low stiffness or elastic modulus. Therefore, traditional RC link-slab with a high reinforcement ratio is neither reasonable for the structural demands nor eco-efficient when compared with ECC link-slabs that demonstrate excellent crack width controllability, durability, ultra-high toughness, and desirable stiffness and elastic modulus [20].

Coupling beams are valuable seismic elements for high-rise buildings. Usually, they can be made of RC with complex steel reinforcement cages and a high reinforcing ratio. When ECC entirely replaces concrete, the steel reinforcement can be simplified and the cost can be reduced [10]. This is because ECC demonstrates much higher ductility, tensile strength, and toughness than ordinary RC and can partially share the tensile stress that would be originally borne by steel rebars.

Also, the ductile response of ECC to earthquake loads allows it to be used as a damper for seismic-resistant structures, which further improves the damage tolerance and service lives of buildings. Real examples can be found in various towers in Japan as shown in Fig. 11 [10].

UHPFRCC has been used in various civil infrastructures since its invention. Besides the areas where ECC can be used, UHPFRCC with high strength (typically, compressive strength > 150 MPa and tensile strength > 8 MPa), high toughness, and enhanced durability can also be used as filling materials for steel tubes and provide security and blast mitigation [85,86]. Figure 12 shows some examples of bridges made from UHPFRCC in several countries [87].

In conclusion, FRCC is a typical composite material developed by the Performance-Driven Design Approach, i.e., a “top to bottom” approach in MIDCS. We can engineer the performance of this material by combining various matrices (typical cementitious composites and high/ultra-high-strength cementitious composites) and fibers (steel, polymer, and natural plant fibers) [88–90] to meet the demands of structures.

### 3.6 Fiber-reinforced polymer bridge decks

Fiber-reinforced polymer (FRP) is also a kind of composite material attracting increasing attention in construction because of its high strength-to-weight ratio, excellent durability, high eco-efficiency, and easy installation on-site [91,92]. It can be used both as a strengthening material for damaged civil infrastructures and as a structural element for new constructions [93]. Here, we mainly focus on applying FRP on bridge decks to demonstrate the concepts of MIDCS.

#### 3.6.1 Properties of fiber-reinforced polymer

The FRP composite is made of a polymer matrix and fibers. Usually, the polymer matrix functions as a binding agent to bind the fibers. The fibers act as tension-bearing elements to improve the toughness, elastic modulus, and ductility of the brittle matrix [91,92]. The fibers and polymers used for FRP composites vary based on the end-use demands of structures. For example, carbon fibers are usually used to produce FRP with excellent mechanical properties (such as tensile strength, elastic

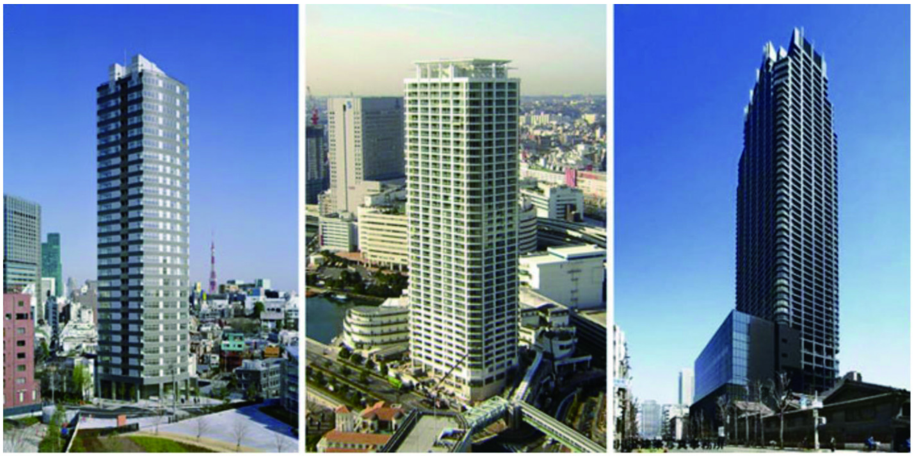

(a) (b) (c)

**Fig. 11** Various buildings with ECC coupling beams in Japan: (a) 27 story Glorio-Tower; (b) 41 story Nabule Yokohama Tower; (c) 60 story Kitahama Tower [10] (Reprinted by permission from Springer Nature Customer Service Centre GmbH: Springer Nature, Springer eBook, Applications of Engineered Cementitious Composites (ECC), Li V C, 2019).

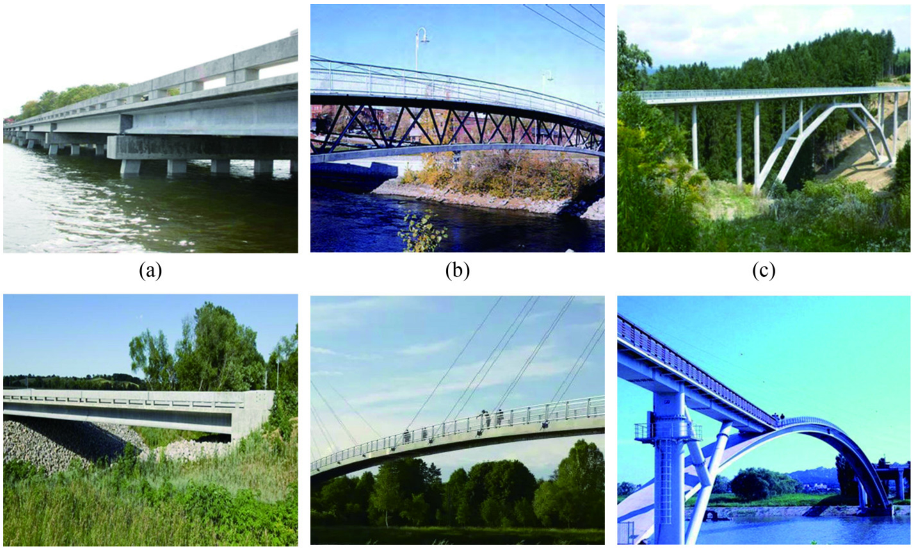

(a) (b) (c)

(d) (e) (f)

**Fig. 12** Examples of UHPFRCC bridges: (a) Cat Point Creek Bridge in the United States; (b) Sherbrooke Overpass in Canada; (c) Wild Bridge in Austria; (d) Mars Hill Bridge in the United States; (e) Celakovice Pedestrian Bridge in the Czech Republic; (f) Peace Bridge in South Korea [87] (Reprinted from Construction and Building Materials, 186, Zhou M, Lu W, Song J, Lee G C, Application of ultra-high performance concrete in bridge engineering, 1256–1267, Copyright 2018, with permission from Elsevier).

modulus, and compressive strength) for strengthening damaged structural elements under a complex stress state [94,95]. Glass fibers are used to create FRP with good corrosion-resistance for marine constructions [96]. Natural fibers (such as sisal, hemp, and wood) make the manufacture of FRP materials and structures more sustainable [97,98]. The polymer matrix most commonly uses epoxy, vinyl ester, or polyester thermosetting plastic for convenient fabrication and installation of FRP elements on-site, as well as easy bonding with other structures [91,92]. This manufacturing approach ensures the FRP can be manufactured in various forms, such as sheets, shapes, wires, etc., to meet the various needs of structures, as shown in Fig. 13 [99]. These advantages of FRP have attracted a great deal of attention in the field of bridge engineering in the past few years. Light-weight, high-strength FRP bridge decks have started to replace traditional RC or steel decks [91–93].

### 3.6.2 Towards high-performance bridge decks

A bridge deck is an important structural element designed to transfer loads transversely to supports. Thus, a bridge deck should have enough strength, elastic modulus, and stiffness to bear external loads. Meanwhile, its deflection should be controlled to avoid possible delamination damages [91,92]. Bridge decks also need to be highly durable against environmental attacks, especially for

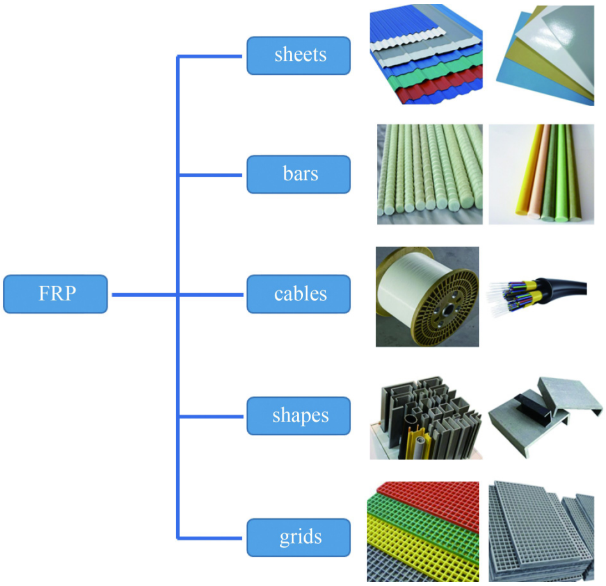

**Fig. 13** FRP products for civil infrastructures [99].

those operating in a marine environment. Chloride-induced steel corrosion and concrete cracking will greatly reduce the designed service life of the bridge and cause a large economic loss. The bridge deck should also be easily installable on-site, whether a new bridge is being built or an existing bridge is being refurnished. More details about the requirements for building a new bridge and refurbishing an existing bridge and their priorities are listed in Table 5 [93]. Due to these demands, FRP composite is a more promising material for high-performance bridge decks than RC or steel.

At present, commercially available FRP bridge decks can be classified into two categories based on their fabrication process: sandwich panels and adhesively bonded pultruded shapes [91,92]. Both categories of FRP products can use standardized manufacturing off-site and modular assembly on-site, allowing for better quality control [91,92].

A typical sandwich panel consists of two strong and stiff face sheets and a low-density core between the two face sheets. The core can be thin-walled cellular FRP materials (honeycomb, corrugated or sinusoidal core), stiff foam, or a hybrid of them, as shown in Fig. 14 [100]. The core material bonds the two stiff face sheets to achieve a composite action, and its low density reduces the dead load of the bridge deck. The stiff face sheet is usually made of E-glass mats or rovings infused with a polyester or vinyl ester thermosetting resin to ensure enough stiffness to transfer the load without excessive flexural deflection. E-glass can be replaced by carbon fiber with a high elastic modulus if the flexural stiffness and deflection need more stringent control, although this will increase costs [91,92].

Sandwich bridge decks can be fabricated off-site through hand layup, or vacuum-assisted resin transfer molding (VARTM). The first approach is an old and labor-intensive method that is more useful for low-volume production. In contrast, the second approach is a semi-mechanized method used for standardized manufacturing, as discussed above. In the course of these fabrication processes, the volume, parameters, and orientations of fibers can be controlled to produce sandwich panels with specific depths, sizes, spans, and even orthotropic structural properties. The sandwich panels are usually connected on-site to form the whole bridge deck using adhesively bonded tongue and groove connections or mechanical shear connectors. Then, the entire bridge deck can be joined to the girders by bolted connections or shear-stud connections with or without composite action with the underlying girders. At the same time, these connections are designed to transfer loads efficiently to ensure all the panels can work together [92].

Adhesively bonded pultruded shapes are widely used to assemble bridge decks because of their well-established assembly processes. Usually, the pultruded decks are unidirectional decks with a constant depth to minimize costs. They are assembled to be aligned transverse to the traffic direction through adhesive bonding (gluing pultruded shapes off-site or pultruded shapes to form the bridge deck on-site) [92]. E-glass fiber is commonly used for producing pultruded shapes because of its relatively low cost. The pultruded decks can be engineered to resist in-plane shear deformation by orienting the fibers at a ±45° angle [92]. Although the pultruded decks have more shear stiffness than the sandwich decks, they have smaller flexural stiffness. Therefore, the bridge deck span should be carefully designed (< 3 m) when using the pultruded decks [92].

**Table 5** Priorities of various demands on using FRP to refurbish a damaged bridge and construct a new bridge [93]

| Priority ↓ | refurbishment | new construction |
|---|---|---|
| | ➢ minimize traffic disruption | ➢ low initial costs |
| | ➢ minimize application time | ➢ low maintenance costs |
| | ➢ low initial costs | ➢ short construction time |
| | ➢ high long-term performance | ➢ minimize traffic disruption |
| | ➢ low maintenance costs | ➢ minimize life-cycle costs |
| | | ➢ minimize environmental impact |

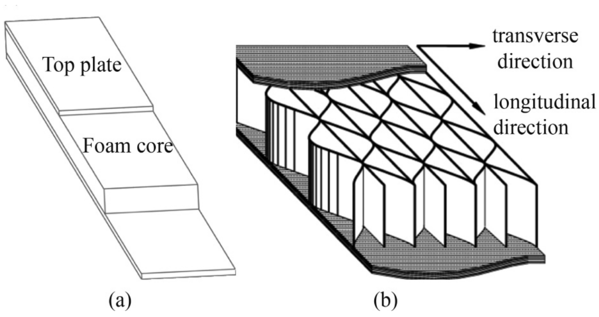

**Fig. 14** Sandwich bridge decks: (a) foam core FRP; (b) sinusoidal core FRP [100] (Reprinted from Composite Structures, 92(7), Chen A, Davalos J F, Strength evaluations of sinusoidal core for FRP sandwich bridge deck panels, 1561–1573, Copyright 2010, with permission from Elsevier).

In summary, FRP bridge decks made from sandwich panels or adhesively bonded pultruded shapes are good examples of the "top to bottom" approach of MIDCS. They can be fabricated off-site through standardized and mechanized processes and installed on-site through convenient connections. They can also be customized to meet the end-use requirements of bridges by controlling the fiber and polymer properties during the manufacturing process, making them powerful, flexible substitutes for conventional concrete decks. Examples of FRP bridge decks can be seen in Ref. [101].

## 4 Advanced methods for material design and structure construction

Based on current-day practices in civil infrastructures, MIDCS is most commonly used: 1) to develop high-performance structures based on available high-performance building materials; 2) to engineer new building materials with properties needed by structure construction. Additive manufacturing (AM) is now a popular method that links material properties to structure construction to achieve the former. For the latter, composite design is a general method that has been used for quite a few years and will continue to be used to engineer the properties of materials. The bioinspired design has also assisted in developing the functional behaviors and mechanical properties of building materials in recent years. Here, we will briefly review advanced methods (AM and bioinspired design) that connect material design and structure construction, with a special focus on cementitious composites because of their wide use in civil infrastructures.

### 4.1 Additive manufacturing for structure construction

AM is a disruptive technology that combines digitalization and automation in construction [102,103]. Its application greatly compensates for the shortage of skilled laborers, resources, and construction efficiency [104]. The application of large-scale AM with cement-based material ink in civil engineering is also known as 3D printing [103,105,106]. A concise definition of 3D printing technology in construction is "transforming an imagination of a facility, in whole or in part, depicted through a computer model, into a real facility (bridges, highways, buildings, etc.), with least human involvement and most conservation of natural resources" [102]. However, more than just cement-based materials can be used as ink for AM or 3D printing processes. Geopolymer-based materials [107,108], polymer materials [109–111], fiber-reinforced composites [80,112,113], and even metals [114–116] can be processed through AM or 3D printing. In most cases, 3D printing is a process where ink material is deposited, joined, or solidified (hardened) layer-by-layer under computer control to create a 3D object. 3D printing of concrete (3DPC) is usually an easy production process not limited by formwork and vibration processes. Also, construction wastes, costs, and time can be greatly reduced with 3DPC [104]. However, it should also be noted that these methods put stricter demands on cement-based materials' workability, setting and hardening time, extrudability, buildability, etc. [102,117,118]. Although some of these properties can be controlled and designed through various chemical and mineral admixtures or rheological methods to meet printable requirements [102], we are still far from the mass production of 3DPC and its widespread application in civil infrastructures. Still, AM or 3D printing technology is a promising method for creating structures with any form we desire. Some examples of 3DPC structures can be found in Fig. 15 [104,119]. Most of them also have appealing aesthetic functions in addition to their structural functions.

### 4.2 Bioinspired material design

Following examples available in nature, we can customize the functional behavior and mechanical properties of materials to meet the requirements of structures. Typical inspirations from nature are from either plants or animals [120]. Here, we give examples of wood (timber) (for functional demands) [121] and nacre or sea urchin spine (for mechanical demands) [122,123] inspired cement with enhanced properties.

Wang et al. designed a wood-inspired cement with high strength and multifunctionality based on the ice-templating technique [121]. This new cement is anisotropic, similar to natural wood, and is lightweight but also high strength. This wood-like cement also has lower thermal conductivities at its transverse profile and good water permeability along its vertical direction. Thus, this wood-inspired cement can be used to achieve the thermal and sound insulation functions of structures that need high strength to mass ratio at a low cost.

There is also cement with high tensile strength and

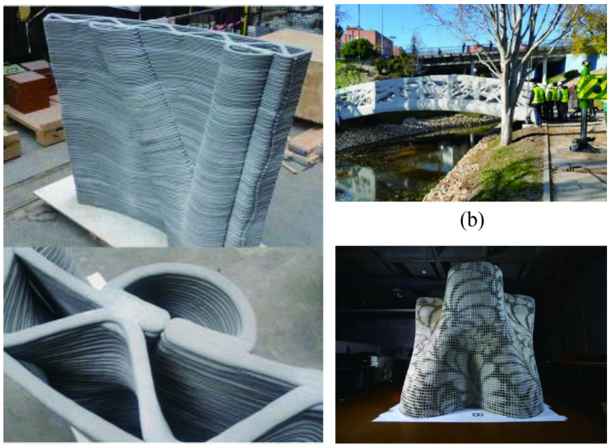

(a) (b) (c)

**Fig. 15** 3DPC structures: (a) 3DPC wall [119] (Reprinted from Materials & Design, 100, Gosselin C, Duballet R, Roux P, Gaudillière N, Dirrenberger J, Morel P, Large-scale 3D printing of ultra-high performance concrete—A new processing route for architects and builders, 102–109, Copyright 2016, with permission from Elsevier); (b) 3DPC arch bridge [104] (Reprinted from Composites. Part A: Applied Science and Manufacturing, 125, Zhang J, Wang J, Dong S, Yu X, Han B, A review of the current progress and application of 3D printed concrete, 105533, Copyright 2019, with permission from Elsevier); (c) 3DPC Bloom Pavilion at UC Berkeley.

toughness inspired by the layered structure of the nacre. By using calcium−silicate−hydrate (C−S−H, the main hydration product of cement) as the “brick” and polymers as the “mortar”, the nacre-inspired cement presents a ductile nature rather than the typical brittle nature of cement. This is usually achieved by incorporating various fibers [122]. A similar bioinspired strategy can also be found in ref. [123]. The authors designed the C−S−H with a meso-crystal structure inspired by sea urchin spine, creating an elastic cement-based material. These ductile and elastic materials can meet the demands of today’s high-performance structures in ways ordinary cement cannot.

## 5 Unified strength-durability design theory

Concrete structures are usually designed to bear mechanical loads as well as be tolerant of various environmental conditions. The mechanical loads and environmental factors degrade the properties of concrete structures with time. However, traditional codes and standards usually treat the mechanical parameters of materials and structures as constant values, which will overestimate the service lives of concrete structures under harsh environmental conditions. Since the 1990s, durability issues have attracted increasing attention because of more durability-related damages to concrete structures. Engineers simplify the durability issue by adjusting the concrete cover thickness for different environmental conditions in their current practice. Thus, there is no scientific or quantitative formula for measuring the environmental factors, and the dynamically changed mechanical properties of materials and structures induced by environmental conditions are not considered in codes and standards.

In a national research project started in 2009 in China, “Basic study on environmentally friendly contemporary concrete”, the load-carrying capability-durability unified service life design theory was first proposed to develop a new and scientific design philosophy where the safety, durability, serviceability, and sustainability of concrete structures can be considered in a unified way [124]. The new theory must resolve two fundamental issues that the current codes and standards do not address. The first issue is to quantitatively measure the environmental factors and combine them with the mechanical loads. The second one is to consider the time-dependent properties of materials and structures carefully.

In detail, to address the first issue, this theory has developed a feasible method that can convert the environmental factor to equivalent stress based on thermodynamics and porous media theory. Take an example of the volume elements of cement-based materials as shown in Fig. 16 [124]: these elements bear a mechanical load in the cement pore solution environment. Based on the thermodynamics and virtue energy calculation, the chemical reactions happening in the pore solutions can be converted into strain or stress. Thus, this theory can quantitatively couple the environmental factors with the mechanical loads by superposing the two stresses (environment factors-induced stress and mechanical loads-induced stress). In this way, the design processes can still follow the existing stress analysis-based codes and standards.

For the second issue, one approach is to determine the deterioration mechanisms of materials and structures under the combined effects of environmental factors and mechanical loads through advanced experimental methods and multi-scale simulations [125,126]. Then, we can get the time-dependent properties of materials and structures and predict the performance of concrete structures as a function of time, as shown in Fig. 17 [124].

Such a unified design method must consider material properties and structural performance through design, construction, service, and maintenance. It is a typical designer-centered multiple disciplinary and holistic approach and considers the entire life cycle of buildings and civil infrastructures. It reflects the nature and philosophy of MIDCS.

## 6 Summary and further research

MIDCS is a classic topic that runs through the

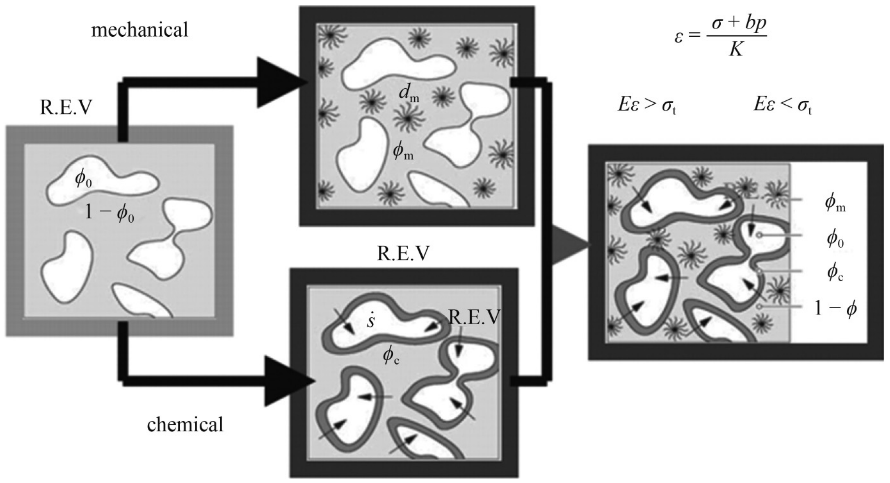

**Fig. 16** Conversion of chemical influence to mechanical effect [124] (with permission).

development history of civil infrastructure. In the early days of civil engineering, we focused mainly on naturally available materials, building the structures according to the properties of those materials. It was a regime of structure construction driven by material properties. Now, with rapid advances in technology, we can alter the microstructure of our materials to improve their performance according to the end-use requirements of the structure. Now, the requirements of structures are the driving force for the design of new building materials.

Today, high-performance structures have been widely developed to make up for the shortage of space caused by population growth. MIDCS is a promising method that combines advanced material design methods and structural construction technologies to meet both material requirements and structural requirements. It will play an important role in continuing the development of high-performance structures in the future. MIDCS is also an approach that will change over time. New avenues of exploration will continue guiding the material design and structure construction. Therefore, future research and applications of MIDCS should explore the following three areas.

1) High-performance materials and structures. Traditionally, the label of “high-performance materials” usually refers to materials with high strengths. But historical practice shows that durability should also be included when considering what high-performance is. On one hand, structures are subjected to mechanical and environmental loads during their service life, making high strength an important property. On the other hand, the environment will cause structures to change in terms of their mechanical properties [124]. This is especially true for high-performance materials and structures that use them, whose design life is usually longer than typical structures. Thus, durability is also an important part of MIDCS.

2) Smart materials and structures. The application of nanotechnology in civil engineering is the gateway to smart materials and structures. We can add various

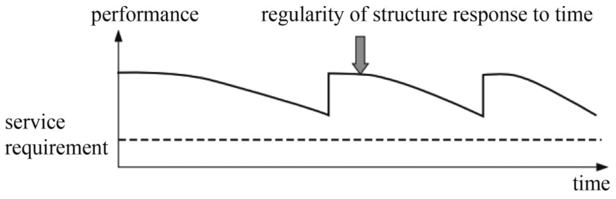

**Fig. 17** Regularity of concrete structure performance as a function of time [124] (with permission).

functions to cement-based materials through nano-modification, such as self-healing, self-cleaning, and self-sensing [10,127]. As a result, structures that use smart materials can give smart responses to external stimuli, which can greatly benefit our daily lives and extend the service lives of structures.

3) Sustainable materials and structures. As one of the most widely used building materials, the cement-based materials, are not as friendly to the environment as they are to civil infrastructures. Cement manufacture accounts for nearly 7% of global carbon dioxide emission and consumes a large amount of energy [128,129]. Therefore, the development of ecologically efficient cement or other green building materials based on life cycle assignment (LCA) and the reuse of construction waste are effective ways to reduce the construction industry’s carbon footprint. MIDCS should also consider this sustainability aspect.

**Acknowledgements** This work was supported by the Science and Technology Development Fund, Macao SAR (0083/2018/A2); Multi-Year Research Grant (MYRG2019-00135-IAPME), Research & Development Grant for Chair Professor (CPG2020-00002-IAPME).

**Open Access** This article is licensed under a Creative Commons Attribution 4.0 International License (https://creativecommons.org/licenses/by/4.0/), which permits use, sharing, adaptation, distribution and reproduction in any medium or format, as long as you give appropriate credit to the original author(s) and the source, provide a link to the Creative Commons licence, and indicate if changes were made. The images or other third party material in this article are included in the article’s Creative

Commons licence, unless indicated otherwise in a credit line to the material. If material is not included in the article’s Creative Commons licence and your intended use is not permitted by statutory regulation or exceeds the permitted use, you will need to obtain permission directly from the copyright holder. To view a copy of this licence, visit http://creativecommons.org/licenses/by/4.0/.
